# Engineering *Geobacillus thermodenitrificans* to introduce cellulolytic activity; expression of native and heterologous cellulase genes

**DOI:** 10.1186/s12896-018-0453-y

**Published:** 2018-06-27

**Authors:** Martinus J. A. Daas, Bart Nijsse, Antonius H. P. van de Weijer, Bart W. A. J. Groenendaal, Fons Janssen, John van der Oost, Richard van Kranenburg

**Affiliations:** 1 Laboratory of Microbiology, Wageningen University, Stippeneng 4, 6708 WE Wageningen, The Netherlands; 2 Laboratory of Systems and Synthetic Biology, Wageningen University, Stippeneng 4, 6708 WE Wageningen, The Netherlands; 3 Corbion, Arkelsedijk 46, 4206 AC Gorinchem, The Netherlands

**Keywords:** *Geobacillus*, Metagenome, β-Xylosidase, Cellulase, CBP

## Abstract

**Background:**

Consolidated bioprocessing (CBP) is a cost-effective approach for the conversion of lignocellulosic biomass to biofuels and biochemicals. The enzymatic conversion of cellulose to glucose requires the synergistic action of three types of enzymes: exoglucanases, endoglucanases and β-glucosidases. The thermophilic, hemicellulolytic *Geobacillus thermodenitrificans* T12 was shown to harbor desired features for CBP, although it lacks the desired endo and exoglucanases required for the conversion of cellulose. Here, we report the expression of both endoglucanase and exoglucanase encoding genes by *G. thermodenitrificans* T12, in an initial attempt to express cellulolytic enzymes that complement the enzymatic machinery of this strain.

**Results:**

A metagenome screen was performed on 73 *G. thermodenitrificans* strains using HMM profiles of all known CAZy families that contain endo and/or exoglucanases. Two putative endoglucanases, GE39 and GE40, belonging to glucoside hydrolase family 5 (GH5) were isolated and expressed in both *E. coli* and *G. thermodenitrificans* T12. Structure modeling of GE39 revealed a folding similar to a GH5 exo-1,3-β-glucanase from *S. cerevisiae*. However, we determined GE39 to be a β-xylosidase having pronounced activity towards *p*-nitrophenyl-β-d-xylopyranoside. Structure modelling of GE40 revealed its protein architecture to be similar to a GH5 endoglucanase from *B. halodurans*, and its endoglucanase activity was confirmed by enzymatic activity against 2-hydroxyethylcellulose, carboxymethylcellulose and barley β-glucan. Additionally, we introduced expression constructs into T12 containing *Geobacillus* sp. 70PC53 endoglucanase gene *celA* and both endoglucanase genes (*M1* and *M2*) from *Geobacillus* sp. WSUCF1. Finally, we introduced expression constructs into T12 containing the *C. thermocellum* exoglucanases *celK* and *celS* genes and the endoglucanase *celC* gene.

**Conclusions:**

We identified a novel *G. thermodenitrificans* β-xylosidase (GE39) and a novel endoglucanase (GE40) using a metagenome screen based on multiple HMM profiles. We successfully expressed both genes in *E. coli* and functionally expressed the GE40 endoglucanase in *G. thermodenitrificans* T12. Additionally, the heterologous production of active CelK, a *C. thermocellum* derived exoglucanase, and CelA, a *Geobacillus* derived endoglucanase, was demonstrated with strain T12. The native hemicellulolytic activity and the heterologous cellulolytic activity described in this research provide a good basis for the further development of *G. thermodenitrificans* T12 as a host for consolidated bioprocessing.

**Electronic supplementary material:**

The online version of this article (10.1186/s12896-018-0453-y) contains supplementary material, which is available to authorized users.

## Background

Lignocellulosic biomass is considered a potential alternative to fossil resources as substrate for biofuels and biochemicals. Although lignocellulosic biomass itself is cheap, saccharification of this substrate is costly due to the variety and amounts of enzymes needed for its conversion. In order to reduce costs of this conversion, Lynd et al. [1] proposed consolidated bioprocessing (CBP) as the most promising solution. CBP requires an organism capable of saccharolytic enzyme production and fermentation of the released sugars. Even if CBP could not be completely achieved, improving hemicellulolytic and cellulolytic activity will be economically attractive.

*Clostridium thermocellum* is considered an excellent candidate for CBP due to the production of a variety of cellulosic enzymes and its thermophilic nature [2]. The use of a thermophile offers several advantages like reduced contamination risk, reduced substrate viscosity and reduced energy demand for cooling [3]. The downside of *C. thermocellum* is that it is not able to ferment C5 sugars released from the lignocellulosic substrate and thus requires extensive engineering of metabolic pathways or the use of co-culturing with pentose utilizing microbes [4–6].

Alternatively, a candidate CBP organism can be derived from the genus *Geobacillus*. Species from this genus are thermophilic, facultative anaerobes that are able to degrade and metabolize hemicellulose [7–9]. For example, *Geobacillus thermodenitrificans* strain T12 has an extensive hemicellulose utilization locus that also includes the capacity to degrade pectin [10]. Unlike *C. thermocellum,* most *Geobacillus* strains are not able to efficiently degrade cellulose, even when isolated from microcrystalline cellulose or composted plant biomass [11, 12]. In the majority of isolates β-glucosidases are present, but endo and exoglucanases are missing. Several isolates with endoglucanase activity have been reported, however, the endoglucanase CelA from *Geobacillus* sp. 70PC53 is to date the only characterized true cellulolytic enzyme native to *Geobacillus* spp. [11, 13]. In a recent study on the isolation of *Geobacillus* strains we showed that several strains of *G. thermodenitrificans* were able to grow on carboxymethyl cellulose, and for some of these strains clear degradation of cellulose was demonstrated by using the Congo red assay [12]. Taken together, these findings demonstrate that genes encoding cellulolytic enzymes are present across the genus *Geobacillus*, although so far only endoglucanases have been found.

An approach to overcome the hurdle of cellulose conversion is to engineer the required cellulose encoding genes into a suitable host of the genus *Geobacillus* [14]. The expression of a heterologous endoglucanase (WP_010885255.1) from *Pyrococcus horikoshii* in *G. kaustophilus* HTA26 enabled this mutant strain to degrade carboxymethylcellulose and filter paper [15]. However, for complete hydrolysis of cellulose both endo and exoglucanases are required and the HTA426 strain lacks genes required for hemicellulose conversion, making it less suited for CBP [16].

Heterologous production of *C. thermocellum* cellulases has been demonstrated in *Bacillus subtillis* [17]. Here, the production of CelK (reducing end exoglucanase; GH 9) and CelS (non-reducing end exoglucanase; GH48) was demonstrated by the clearing zones of mutant colonies on CMC plates. Both exoglucanases described are also highly expressed in *C. thermocellum* when grown on cellulosic and lignocellulosic substrates and are therefore expected to be of great importance for the cellulolytic activity of *C. thermocellum* [18–20].

In this study, we screened the metagenome of 73 *Geobacillus thermodenitrificans* isolates for novel endo and exoglucanases. Subsequently, three *Geobacillus* derived endoglucanases were expressed in *E. coli* and *G. thermodenitrificans* T12. To complement the cellulolytic activity of strain T12 we also introduced an endoglucanase (*celC*) and two exoglucanases (*celK* and *celS*) from *C. thermocellum*.

This is the first study on the expression of a full set of cellulolytic enzymes in *Geobacillus* and thereby provides new insights in the applicability of members of this genus as potential hosts for consolidated bioprocessing.

## Methods

### Media, strains, primers and constructs

Cellulolytic Thermophile Vitamin Medium (CTVM; based on [7, 21–23]) contained per liter: 8.37 g MOPS; 1 g NH_4_Cl; 3 g NaCl; 1.50 g Na_2_SO_4_; 0.08 g NaHCO_3_; 1 g KCl; 1.8 g MgCl_2_ × 6H_2_O; 0.30 g CaCl_2_ × 2H_2_O. pH was set to 6.6 at room temperature and the medium was autoclaved for 20 min at 121 °C, after which 1 mL K_2_HPO_4_ (250 g/L; pH 6.6)_,_ 10 mL filter sterile 100× metal mix and 1 mL filter sterile 1000× vitamin solution were added. 100× metal mix contained per liter: 1.60 g MnCl_2_ × 6H_2_O; 0.1 g ZnSO_4_; 0.2 g H_3_BO_3_; 0.01 g CuSO_4_ × 5H_2_O; 0.01 g Na_2_MoO_4_ × 2H_2_0; 0.1 g CoCl_2_ × 6H_2_O; 0.7 g FeSO_4_ × 7H_2_O; 5 g CaCl_2_ × 2H_2_O; 20 g MgCl_2_ × 6H_2_O. 1000× vitamin mix contained per liter: 0.1 g thiamine; 0.1 g riboflavin; 0.5 g nicotinic acid; 0.1 g pantothenic acid; 0.5 g pyridoxamine, HCl; 0.5 g pyridoxal, HCl; 0.1 g D-biotin; 0.1 g folic acid; 0.1 g *p*-aminobenzoic acid; 0.1 g cobalamin.

LB2 contained per liter: 10 g tryptone (Oxoid), 5 g yeast extract (Roth), 10 g sodium chloride and salts mix consisting of 1 g NH_4_Cl; 3 g NaCl; 1.50 g Na_2_SO_4_; 0.08 g NaHCO_3_; 1 g KCl; 1.8 g MgCl_2_ × 6H_2_O; 0.30 g CaCl_2_ × 2H_2_O. pH was set to 6.6 at room temperature and the medium was autoclaved for 20 min at 121 °C, after which 10 mL K_2_HPO_4_ (250 g/L) was added.

Minimal Media (MM) contained per liter: 0.52 g K_2_HPO_4_; 0.23 g KH_2_PO_4_; 0.5 g NH_4_NO_3_ (MMy) or 0.3 g NH_4_Cl (MMy+). After autoclaving, 1 mL of the following 1,000× concentrated sterile stocks were added: Nitrilotriacetic acid (200 g/L); MgSO_4_ × 7H_2_O (145.44 g/L); CaCl_2_ × 2H_2_O (133.78 g/L); FeSO_4_ × 7H_2_O (11.12 g/L).

For CTVMy/MMy medium, 0.5 g/L yeast extract (Roth) was added to the medium and CTVMy+/MMy + contains 5 g/L yeast extract (Roth).

Glycerol stocks of cultures were made by adding 500 μl sterilized 60% glycerol to 1.5 mL culture, in a 2 mL cryogenic vial (Corning). Stocks were stored at − 80 °C.

In all plate and tube cultures, carbon substrates were used in a concentration of 10 g/L unless stated otherwise. For plate cultures, 5 g/L gelrite (Roth) was added.

*Geobacillus thermodenitrificans* T12 was isolated from compost [12]. *E. coli* DH5α and *E. coli* TG-90 were used for DNA manipulation and *E. coli* BL21(DE3) and *G. thermodenitrificans* T12 were used for protein production. *E. coli* DH5α and BL21(DE3) were grown at 37 °C in Luria-Bertani (LB) medium and *E. coli* TG-90 was grown in LB medium at 30 °C. Wild-type *G. thermodenitrificans* T12 was grown at 65 °C and at 55 °C when harbouring plasmid DNA to maintain plasmid replication.

### DNA isolation, sequencing and assembly

All strains were grown in LB2 media at 65 °C in a rotary shaker at 150 RPM. Genomic DNA was isolated from 10 mL of logarithmic growing cultures of an OD_600_ of approximately 1.00 AUs by using the MasterPure™ Gram Positive DNA Purification Kit (Epicentre, Madison, Wisconsin, USA) according to manufacturer’s protocol. Genomic DNA was then pooled and sent for sequencing by the company Baseclear B.V. (Leiden, The Netherlands). Paired-end sequence reads were generated using the Illumina HiSeq2500 system. FASTQ sequence files were generated using the Illumina Casava pipeline version 1.8.3. The initial quality assessment was based on data passing the Illumina Chastity filtering. Subsequently, reads containing adapters and/or PhiX control signal were removed using an in-house filtering protocol. Reads were aligned to the genome of *G. thermodenitrificans* T12, a strain known to contain no functional cellulases, with Bowtie2 v2.2.4 (parameters: –local --no-mixed –no-discordant) [24]. The unaligned reads were assembled with Ray v2.3.1 with a kmer set to 81. The same unaligned reads were aligned to the assembled scaffolds with Bowtie2 v2.2.4 and converted to sorted BAM files with Samtools v1.1 [25]. The sorted BAM files were used as an input for Pilon v.1.10 [26] for automatic error correction resulting in 2616 scaffolds with a total of 28,814,104 bp and a n50 of 41,189 bp with average coverage of 715.

### Selection and sequence analysis of metagenome putative cellulases

**Fig. 1:**
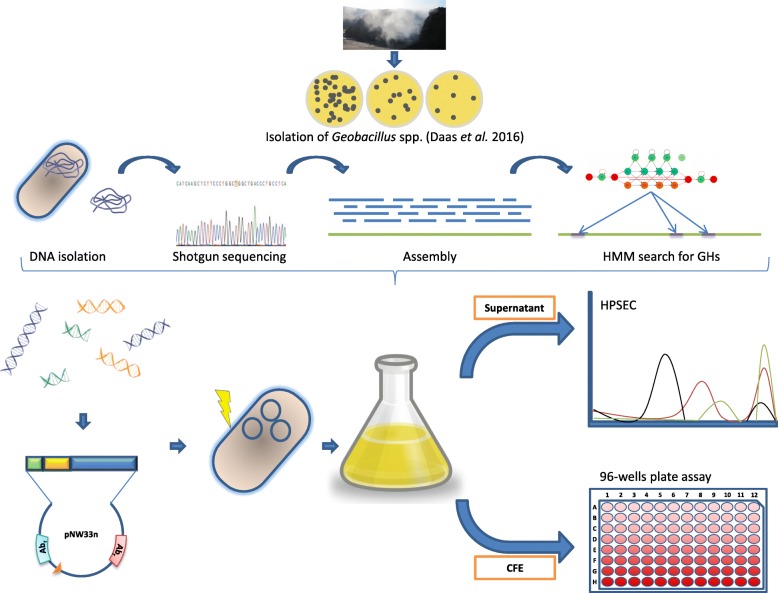
Overview of the workflow for detecting novel cellulases from a *G. thermodenitrificans* metagenome. From compost a total of 73 *G. thermodenitrificans* strains were isolated of which the genomic DNA was pooled and subjected to shotgun sequencing. By screening the assembled metagnome data on proteins matching against HMM profiles of all known glycoside hydrolase families that contain endoglucanase and/or exoglucanases we identified several novel putative cellulases. The genes encoding these putative cellulases were used to create expression constructs in both *E. coli* and *G. thermodenitrificans* T12. The supernatant and CFE of these cultures were then subject to activity assays against a variety of substrates

Prodigal v2.6.1 [27] was used for gene prediction with the *G. thermodenitrificans* T12 genome as a training set. The predicted proteins were used as input for hmmsearch v3.1b1 [28] to identify possible cellulases with Hidden Markov models (HMMs) from PFAM [29] and dbCAN [30] of all known glycosyl hydrolase (GH) families that contain endoglucanases and/or exoglucanases: GH 1, 5, 6, 7, 8, 9, 10, 11, 12, 26, 44, 45, 48, 51, 74 and 124 (accession numbers resp. PF00150.16, PF01341.15, PF00840.18, PF01270.15, PF00759.17, PF00331.18, PF00457.15, PF01670.14, PF02156.13, PF12891.5, PF02015.14, PF02011.13). Models for GH 51, 74 and 124 were obtained from dbCAN. Proteins fitting the models were functionally annotated with BlastP v2.6.0+ using the TrEMBL v2017_1 protein database. A schematic overview of the procedure is given in Fig. 1.

Initial protein function was based on best hits in the TrEMBL v2017_1 protein database, and then manually filtered on their predicted function. The remaining selection (Table 1) was subject to further sequence analysis, which involved protein model prediction by Phyre2 [31], determination of conserved active site residues and signal peptide prediction by SignalP4.1 [32].

**Table 1:** Selection of putative endoglucanases retrieved from the metagenome data and their closest orthologs from the TREMBL v080914 protein database

Query	E-value	Bitscore	Hit-Gene ID	AA identity (%)	Protein names	Organism
GE32	0	2673	A0A0Q1EJD2	100	GEPA3_1816	*Geobacillus* sp. PA-3
GE33	0	2640	A0A0Q1EJD2	98	GEPA3_1816	*Geobacillus* sp. PA-3
GE39	0	2559	A0A0Q1EJD2	96	GEPA3_1816	*Geobacillus* sp. PA-3
GE40	0	1691	E6TRT3	58	LPXTG-motif cell wall anchor domain protein	*Bacillus cellulosilyticus* (DSM 2522)

### Expression of recombinant putative endoglucanases GE39 and GE40

**Fig. 2:**
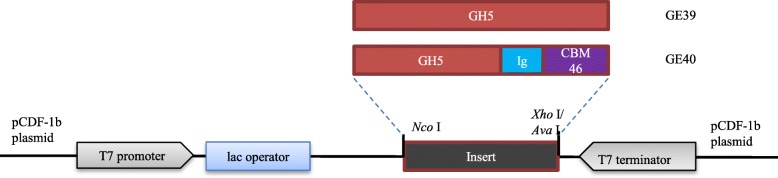
Expression constructs of two putative endoglucanase encoding genes derived from the *G. thermodenitrificans* metagenome analysis. Gene *GE39* encodes a GH5 catalytic domain and gene *GE40* encodes a GH5 catalytic domain followed by an immunoglobulin-like domain (Ig) and a carbohydrate module of family 46 (CBM). Both genes were expressed in *E. coli* BL21(DE3) using the pCDF-1b plasmid under control of the *lac* operator

Selected putative endoglucanase genes were amplified by high-fidelity PCR using PhusionHF polymerase. The PCR mixes contained 10 μl Phusion HF Buffer, 1 unit of Phusion DNA polymerase (Thermoscientific), 100 μM of dNTPs, 20 ng DNA, 0.2 μM of both the forward primers BG6897 (GCGCCATGGAAATGCTTAAGGTCACTA) for GE39 and BG6899 (GCGCCCATGGAAGACAATAAAGCGTCGGCATAC) for GE40, and the reverse primers BG6898 (CGCCTCGAGTTATAAACTTATACTGGACTGATTTG) for GE39 and BG6900 (CGCCTCGAGCTACTTTCCGGCCATCTTCAA) for GE40. MilliQ was added to a total volume of 50 μl. PCR products were checked on 1% agarose gels and products were purified by using a GeneJet PCR purification kit (Fermentas). The PCR products of GE39 were digested with restriction enzymes *Nco*I and *Xho*I and purified PCR products of GE40 were digested with restriction enzymes *Nco*I and *Ava*I and subsequently cloned into the pCDF-1b vector (Fig. 2). The recombinant plasmids were introduced to *E. coli* DH5α using heat shock competent cells and then plated on selective media. Plasmids were purified using the GeneJET plasmid miniprep kit according to manufacturer’s protocol and were subsequently introduced to *E. coli* BL21(DE3) for enzyme expression. BL21 strains containing the recombinant endoglucanases were cultured overnight in 10 mL LB medium in a 37 °C rotary shaker at 150 RPM. Next morning, cultures were cooled on ice for 10 min prior to being transferred to 50 mL LB medium supplemented with 50 μg/ml streptomycin and grown at 20 °C under constant agitation at 150 RPM. Expression of the endoglucanases was induced using 0.1 mM isopropyl-β-thioglactopyanoside (IPTG) at an OD600 of approximately 1.00.

### Extraction and characterization of recombinant predicted endoglucanases GE39 and GE40

The induced *E. coli* BL21(DE3) cultures (50 mL) containing the recombinant endoglucanases GE39 and GE40 were collected after 18 h by centrifugation at 4800×*g* for 15 min at 4 °C. The cells were resuspended in 5 mL of 200 mM sodium phosphate buffer (pH 6.00) and disrupted using a French press at 1200 psi. For DNA lysis, DNase I was added (1 mg/mL) to the crude extracts and incubated at room temperature for 15 min. The cell debris was removed by centrifugation at 30,000×*g* for 15 min at 4 °C. The resulting supernatant was filter-sterilized (0.45 μm) to remove remaining cell debris and protein concentrations of the obtained cell free extract (CFE) were determined by the Bradford method using the Bradford Reagent Assay Kit (Sigma) with the bovine serum albumin as the standard [33].

To determine saccharolytic activities of the CFE we used a series of chromogenic substrates in a Glycospot Multi CPH 96-wells filter plate [34] (Glycospot, Frederiksberg C, Denmark). Substrates were activated by the addition of 200 μL activation solution. Centrifugation (2700×*g*, 10 min) was applied to remove the solution followed by a double wash with 100 μL milliQ. The final reaction mixture in each well consisted of 145 μL sodium phosphate buffer (pH 6.0) and 5 μL of CFE. Plates were then sealed using an aluminium adhesive foil (VWR, Radnor, PA, USA) and incubated at 60 °C in a rotary shaker at 180 RPM. After 24 h the reaction mixture was collected in the product plate by centrifugation (2700×*g*, 10 min) and absorbance was measured at 595 nm (blue) and 517 nm (red) using a plate reader (Biotek Instruments Inc., Winooski, VT, USA). Negative controls consisted of sodium phosphate buffer and CFE from an *E. coli* culture containing empty pCDF1b plasmid. The thermostable endoglucanase, CelTM, (Megazyme, Wicklow, Ireland) from *Thermotoga maritima* was used as positive control at a concentration of 1 μg/mL.

To determine exo-activity, we used 3 μg of CFE mixed with 200 μL of a 200 mM sodium phosphate buffer (pH 6.00). The reaction was started by adding 10 μL of 50 mM *p-*nitrophenyl-β-d-xylopyranoside (pNPβX) or *p-*nitrophenyl-β-d-glucopyranoside (pNPβG) (Sigma, St. Louis, MO, USA) and incubated at 60 °C for 10 min. Negative controls consisted of sodium phosphate buffer and CFE from an *E. coli* culture containing empty pCDF1b plasmid. Reaction was stopped by adding 1 mL of 0.5 M bicarbonate and the amount of released pNP was determined by absorbance measurement at 410 nm and subsequently plotting the data against a standard curve generated using pNP as a substrate. One unit (U) of activity was defined as the release of 1 μmol pNP per minute.

### Expression constructs of *Geobacillus* endoglucanases

Linear constructs of the metagenome derived GE40 encoding gene as well as *Geobacillus* endoglucanases *M1* (GI:523426779), *M2* (GI:523426040) and *celA* (GI:214003628) were synthetically manufactured by Bio Basic Inc. (Amherst, NY, USA). Constructs were composed of the P_*upp*T12_ promoter driving expression of the various endoglucanase genes supplemented with the coding sequence for the *Gt*XynA1 (KX962565.1) signal peptide and were separately cloned into the pNW33n vector using restriction enzymes listed in Table 2. Ligation mixes were introduced directly to *G. thermodenitrificans* T12 as previous attempts in cloning the *Geobacillus* constructs to *E. coli* DH5α and TG90 failed to yield correct transformants. Transformation and recovery of the transformed T12 cells was performed as described before with minor modifications [12].Strain T12 was grown O/N in LB2 after which the cells were diluted in 50 mL fresh LB2 medium (OD_600_ = 0.05) in a 250 mL baffled shake flask. This pre-culture was incubated at 65 °C in a rotary shaker at 180 RPM. When OD_600_ reached 0.95, the cells were pelleted by centrifugation (4800×*g*) and washed twice with ice-cold milliQ (50 mL) and twice with 10% glycerol (25 mL and 10 mL, respectively). Competent cells were then aliquoted (65 μL) and incubated with 1 μg plasmid DNA for 2 min. Following electroporation (2 kV, 200 Ω, 25 μF), cells were recovered for 2 h in 1 mL pre-warmed LB2 at 55 °C. Cells were then plated on LB2 agar containing 7 μg/mL chloramphenicol. Colonies were picked after 24 h of growth at 55 °C and plasmid presence and integrity was verified by PCR using FW primers BG3665 (5′- GCTCGTTATAGTCGATCGGTTC-3′), or BG3859 (5′- GTTTGCAAGCAGCAGATTACG-3′) for *celA*, and RV primer BG3664 (5’-AGGGCTCGCCTTTGGGAAG-3′).

**Table 2:** Overview of the different elements of all construct and the restriction enzymes used for the insertion of the construct to the pNW33n plasmid

Gene origin	Construct name	Promoter	Signal peptide	Original protein fragment		Construct length (bp)	Restriction enzymes
				Start (AA)	End (AA)		
*M1* (GI:523426779)	T12-M1	P_*upp*T12_	*Gt*XynA1	1	389	1361	*Acc*65I / *Pst*I
*M2* (GI:523426040)	T12-M2	P_*upp*T12_	*Gt*XynA1	1	359	1263	*Pst*I / *Hind*III
*celA* (GI:214003628)	T12-CelA	P_*upp*T12_	*Gt*XynA1	25	392	1294	*Bsp*HI / *Eco*RI
*GE39*	T12-GE39	P_*upp*T12_	*Gt*XynA1	1	488	1674	*Acc*65I / *Pst*I
*GE40wt*	T12-GE40wt	P_*upp*T12_	GE40	1	538	1732	*Acc*65I / *Bam*HI
*GE40*	T12-GE40	P_*upp*T12_	*Gt*XynA1	28	538	1732	*Acc*65I / *Bam*HI
*celK* (GI:2978566)	T12-CelK	P_*upp*T12_	*Gt*XynA1	28	812	2536	*Acc*65I /*Xba*I
*CelC* (GI:12584559)	T12-CelC	P_*upp*T12_	*Gt*XynA1	1	343	1219	*Bsp*HI / *Eco*RI
*CelS* (GI:145558928)	T12-CelS	P_*upp*T12_	*Gt*XynA1	28	674	2130	*Pst*I / *Hind*III
*CelSH* (GI:145558928)	T12-CelSH	P_*upp*T12_	*Gt*XynA1	28	674	2130	*Pst*I / *Hind*III

Original protein fragment numbers indicate the fragment of protein encoded by the partial gene used to build the different constructs

*AA* amino acid

### *C. thermocellum* cellulases CelC, CelK and CelS

**Fig. 3:**
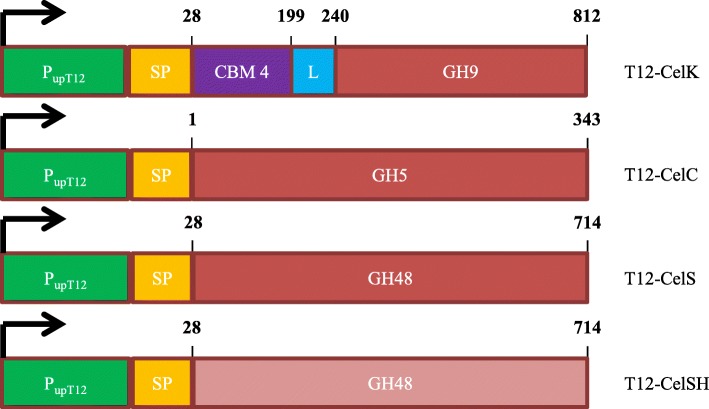
Overview *C. thermocellum* constructs for expression in *G. thermodenitrificans* T12. Numbers indicate start and end amino acid residues of the original cellulases as deposited in the UniprotKB database. Green: promoter sequence derived from the uracil phosphoribosyltransferase gene native to *G. thermodenitrificans T12*, yellow: signal peptide sequence derived from the endoxylanse encoding gene (*GtxynA1*) native to *G. thermodenitrificans* T12, purple: carbohydrate binding domain encoded by *celK* from *C. thermocellum*, blue: linker domain of the *celK* gene from *C. thermocellum*., red: catalytic domains of the indicated genes, orange: *G. thermodenitrificans* codon harmonized sequence encoding the catalytic domain of *celS*

Linear constructs of the genes encoding *C. thermocellum* exoglucanase CelK and endoglucanases CelS and CelC were synthetically manufactured by Bio Basic Inc. (Amherst, NY, USA). Constructs were composed of the P_*upp*T12_ promoter driving expression of the various catalytic domains fused to the carbohydrate binding module of each of the *C. thermocellum* cellulases supplemented with the coding sequence for the *Gt*XynA1 (KX962565.1) signal peptide (Fig. 3). Constructs were separately cloned into the pNW33n vector using restriction enzymes listed in Table 2. Because of the severe mismatch in codon usage between *G. thermodenitrificans* and the gene *celS* from *C. thermocellum*, a codon harmonized variant (*celSH*) was synthesized. The codon landscape and harmonized sequence of *celS* are given in Additional file 1: Table S1. Constructs were separately cloned into the pNW33n vector and ligation mixes were introduced to *E. coli* TG90. Recovery of the transformed TG90 cells was done at 30 °C for 2.5 h in a rotary shaker at 150RPM, as recovery at 37 °C failed to yield correct transformants. Cells were plated on LB agar containing 12.5 μg/mL chloramphenicol. Colonies were picked after 48 h of growth at 30 °C and plasmid presence and integrity was verified by PCR using FW primers BG3665 (or BG3859 for *celC*) and RV primer BG3664. Plasmids containing the correct insert sequence were isolated using JETstar Plasmid Purification MAXI Kit (Genomed, Löhne, Germany) according to manufacturer’s protocol. Purified plasmids were subsequently introduced to *G. thermodenitrificans* T12 as previously described and positive clones were verified by PCR and sequencing of the plasmid insert.

### Endoglucanase activity assays

Cultures (50 mL) of *G. thermodenitrificans* T12 containing the different cellulase constructs were grown in LB2 medium containing 0.5% CMC as substrate. After 18 h of growth at 55 °C a 10 μL sample was taken and spotted on solid LB2 medium containing 0.5% CMC. Plates were incubated for 24 h at 55 °C and subsequently stained for 5 min by flooding the plates with 0.1% Congo red dye followed by destaining with a 1 M NaCl solution for 15 min.

The remainder of the T12 cultures were centrifuged (4800×*g*, 10 min) after which the cell fractions were disrupted using a French press at 1200 psi. The obtained cell lysates were centrifuged (30,000×*g*, 15 min) to obtain clear CFEs. To determine saccharolytic activities of the CFE we used a series of chromogenic substrates in a Glycospot Multi CPH 96-wells filter plate [34] as described above. Negative controls consisted of sodium phosphate buffer and CFE of a T12 culture containing an empty pNW33n plasmid. The thermostable endoglucanase, CelTM, (Megazyme, Wicklow, Ireland) from *Thermotoga maritima* was used as positive control at a concentration of 1 μg/mL.

Cellulolytic activity of the cultures supernatants was analysed by high performance size exclusion chromatography (HPSEC) on an Ultimate 3000 HPLC system (Thermo Scientific, Sunnyvale, CA, USA) equipped with a set of three TSK-gel columns (6.0 mm × 15.0 cm per column) in series (SuperAW4000, SuperAW3000, SuperAW25000, Tosoh Bioscience, Stuttgart, Germany) in combination with a PWX-guard column (Tosoh Bioscience). HPSEC was controlled by the Chromeleon software (Thermo Scientific). Elution took place at 55 °C with 0.2 M sodium nitrate at a flow rate of 0.6 mL/min. The eluate was monitored using a refractive index (RI) detector (Shoko Scientific Co., Yokohama, Japan). Calibration was made by using pullulan series (Polymer Laboratories, Union, NY, USA) with a molecular weight in the range of 0.18–788 kDa.

### Nucleotide sequence accession numbers

Nucleotide sequences of GE32, GE33, GE39 and GE40 were deposited in GenBank with accession numbers MF969097, MF969098, ATG84609 and ATG84593, respectively. The nucleotide sequence of scaffold_9 from *G. thermodenitrificans* T81 has been deposited in GenBank with accession number MF170616.

## Results

### Selection and sequence analysis of metagenome putative cellulases

**Fig. 4:**
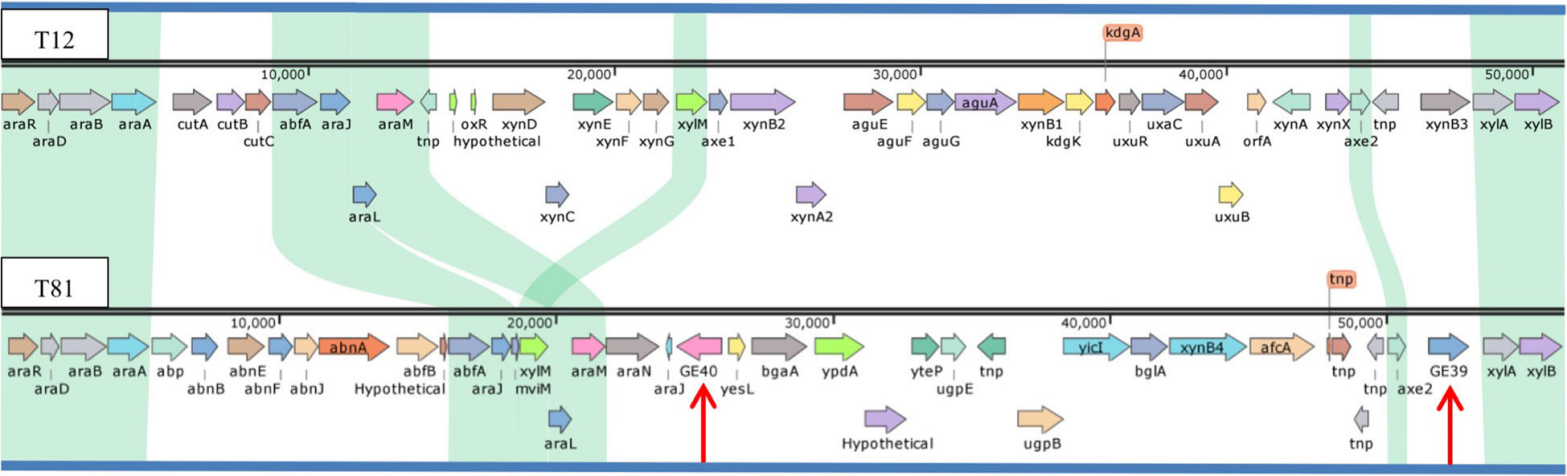
Overview of the partial HUS loci of *G. thermodenitrificans* T12 (top) and scaffold 9 (S9, bottom). Annotations were assigned based on protein sequence blast (BlastP) against the National Center for Biotechnology (NCBI) non-redundant protein database. Comparison between the predicted CDSs of the two HUS loci was performed using a localized BlastP analysis in CloneManager v9.51 (Sci-ed software, Denver, CO, USA). Conserved genes are shaded in green, genes coding for endoglucanase GE40 and β-xylosidase GE39 are indicating by red arrows

**Fig. 5:**
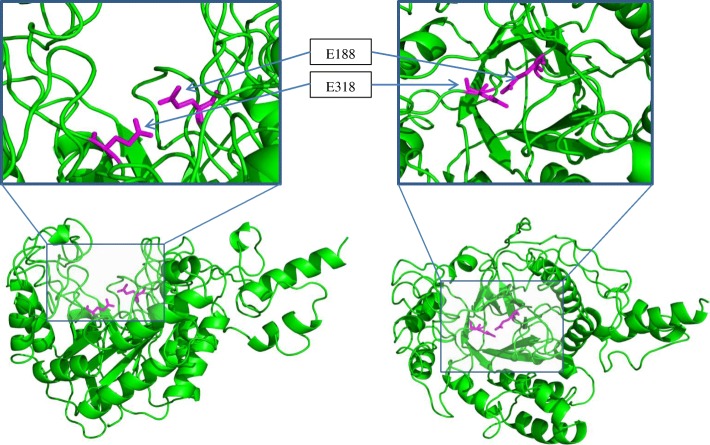
Protein structure of the GE39 endoglucanase protein. The protein model has been predicted using Phyre2 on the basis of 7 templates. 98% of the residues from GE39 were modelled at > 90% confidence. The GE39 protein has a GH5 catalytic domain with active site residues (pink sticks) predicted to be E178 and E296 based on multiple alignment

In a previous study we showed that several strains of *G. thermodenitrificans* out of a collection of 73 isolates were able to grow on carboxymethyl cellulose, and for some of these strains clear degradation of cellulose was demonstrated by using the Congo red assay [12]. As no cellulases are known for *G. thermodenitrificans*, we performed a metagenome sequencing analysis to retrieve potential cellulases. A total of 82 hits for potential endoglucanases or exoglucanases were identified in the metagenome of 73 *G. thermodenitrificans* strains using HMM profiles against all glycoside hydrolase families known to contain endoglucanases and/or exoglucanases. After manual inspection of the best hits with the uniprotKB database, we obtained a final selection of four (GE32, GE33, GE39 and GE40) potential endoglucanases which were subject to further analysis. Three of the four putative endoglucanases, GE32, GE33 and GE39, showed high amino acid sequence identity (> 94%) between each other. Therefore, we assumed these three proteins to have identical activities and we selected GE39 and GE40 for our assays. One scaffold (assigned scaffold 9) contained both the *GE39* and *GE40* genes and was annotated further using BlastP against the NCBI non-redundant protein database. Comparison of this scaffold with the genome of *G. thermodenitrificans* T12 revealed that the two genes reside in the hemicellulose utilization (HUS) locus. Great variation in genetic content was observed between the HUS loci of strain T12 and scaffold 9 (Fig. 4). However, the localization of the *GE39* gene on scaffold 9 is clustered with *xylA* and *xylB* which may suggest a role as a β-xylosidase, in analogy to the XynB3 encoding gene of strain T12. Indeed, the GE39 enzyme shows most amino acid sequence identity (36%) to the characterized β-xylosidase (*Pc*Xyl5) from *Phanerochaete chrysosporium* and contains GH5-family conserved glutamine residues at positions E188 and E318 (Fig. 5).

**Fig. 6:**
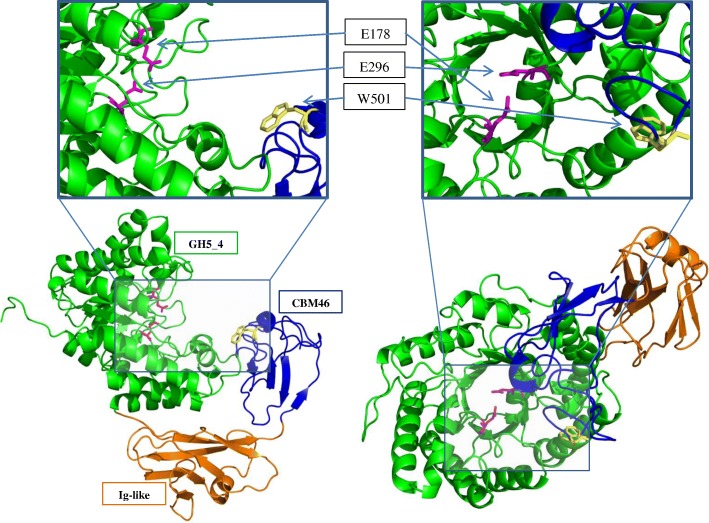
Protein structure of the GE40 endoglucanase protein. The protein model has been predicted on the basis of the crystal structure of a *Bacillus halodurans* endoglucanase (BhCel5b; PDB:4V2X_A). 92% of the residues from GE40 were modelled at > 90% confidence. The GE40 protein contains an N-terminal GH5_4 catalytic domain, an immunoglobulin-like domain and a C-terminal CBM46. Active site residues were to be E178 and E296 (pink sticks). The tryptophan residue at position W501 (yellow stick) was predicted to be involved in ligand binding

For gene *GE40* we could not predict its putative function based on this alignment as we could not relate *GE40* to any of the T12 genes nor to any other *Geobacillus* derived protein from the NCBI non-redundant database. The amino acid sequence of GE40 is closest related (51 and 31% AA sequence identity) to the well characterized GH5 family endoglucanases from *Bacillus halodurans* (*Bh*Cel5b, PDB:4V2X_A) and *Bacillus licheniformis* (*Bl*Cel5b, PDB: 4YZT_A), respectively (Additional file 2: Figure S1). Sequence comparison shows similar protein architecture of both Cel5b proteins and protein GE40. GE40 is comprised of a GH5_4 catalytic domain, an immunoglobulin-like module and a carbohydrate binding module belonging to family 46 (Fig. 6). Deeper analysis of the amino acid sequence of GE40 reveals conserved active site residues of the GH5_4 domain at positions Glu-178 and Glu-296, and a conserved residue active in ligand-binding of the CBM to be Trp-501 (Fig. 6) [35, 36]. The location of Glu-178 and Glu-296 on the C-termini of the fourth and seventh ß-strand respectively, is in accordance with the known position of active site residues in enzymes belonging to the GH5 family [35, 37].

**Fig. 7:**
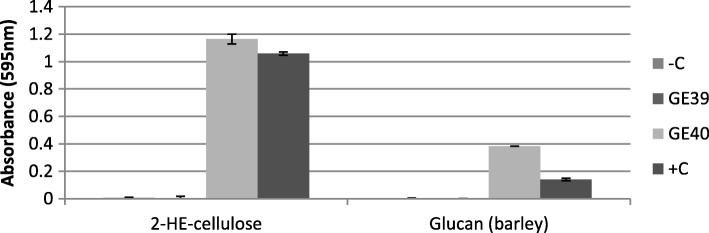
Enzyme activity of CFE from GE39 and GE40 expressing *E. coli* BL21(DE3) cultures. CFE was incubated at a pH of 6.0 on a series of chromogenic substrates and incubated for 24 h at 60 °C in a rotary shaker (180 RPM). Degradation of the substrate by cellulolytic activity was measured by colorimetric measurements of the solubilized chromogenic oligosaccharides at 595 nm

Cellulolytic activity of the GE40 and GE39 proteins produced in *E. coli* was measured using the solubilized fraction of the chromogenic substrates in a Glycospot Multi CPH assay plate (Additional file 3: Table S2). CFE of the GE40 expressing *E. coli* culture showed high activity towards cellulose and barley derived β-glucan. In contrast, CFE of the GE39 producing *E. coli* culture showed no activity to any of the chromogenic substrates (Fig. 7, Additional file 3: Table S2). However, it showed activity towards *p-*nitrophenyl-β-d-xylopyranoside (55 U/mg) along with some side activity towards *p-*nitrophenyl-β-d-glucopyranoside (11 U/mg). We therefore conclude that GE39 is a β-xylosidase.

### *G. thermodenitrificans* cellulolytic activity assays

**Fig. 8:**
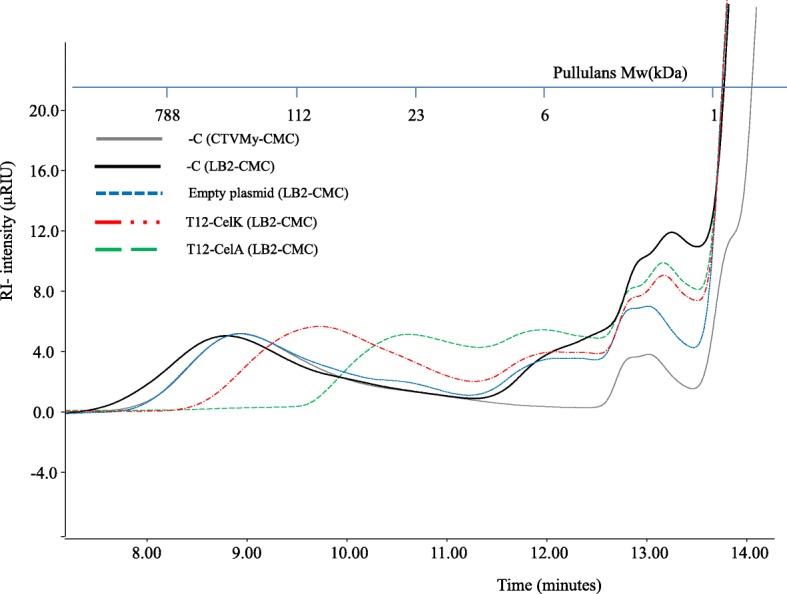
HPSEC analysis of carboxymethylcellulose (CMC) degradation by *G. thermodenitrificans* T12. Cultures were grown for 24 h on LB2 with 1% CMC. The degradation of the cellulose is visualized by the change in molecular weight of the peak eluting between 8 and 9 min. Both cultures expressing CelA (T12-CelA, green) and CelK (T12-CelK, red) show a clear degradation of cellulose in contrast to the empty plasmid control (Empty plasmid, blue). Negative controls (-C) consisting of non-inoculated media show some effect of yeast extract around an elution time of 12–14 min

The GE40 metagenome endoglucanase, the *Geobacillus* endoglucanase encoding genes *M1*, *M2* and *celA*, and the *C. thermocellum* cellulase encoding genes (c*elK, celC and celS*) were used to create expression constructs for *G. thermodenitrificans* T12. The chosen genes from *C. thermocellum* have been reported to be highly expressed in *C. thermocellum* when grown on cellulosic and lignocellulosic substrates and are therefore expected to be of great importance for the cellulolytic activity of *C. thermocellum* [18–20]. *From* these genes, only the catalytic domains and, if present, the carbohydrate binding modules were used and fused with the *Gt*XynA (extracellular endoxylanase) signal peptide and the P_*upp*T12_ promoter sequence (derived from the uracil phosphoribosyltransferase encoding gene of strain T12), which have both successfully been used for heterologous protein production in *G*. *thermodenitrificans* T12 [12]. For endoglucanase GE40, we created two constructs; T12-GE40wt: the native gene under control of the P_*upp*T12_ promoter and T12-GE40: as T12-GE40wt but with the *Gt*XynA1 signal peptide as replacement for the original signal peptide. Also, for the expression of *C. thermocellum* exoglucanase *celS* we created two constructs; one with the original sequence (T12-*celS*) and one with a codon harmonised sequence (T12-*celS*^*H*^). Constructs were introduced to strain T12 using the pNW33n vector and functional activities were tested in vivo using the Congo red assay and HPSEC analysis. Strains harbouring constructs with the *C. thermocellum celC*, *celS* and *celS*^*H*^ genes lack cellulase activity. However, strains harbouring a construct derived from *C. thermocellum celK*, the *Geobacillus celA* or the *GE40* gene showed activity against amorphous cellulose (Additional file 4: Figure S2). This activity was also confirmed by HPSEC analysis for constructs T12-CelK and T12-CelA, indicated by the reduced MW of the CMC peak eluting between 8 and 10 min (Fig. 8). The activity of T12-GE40 was too low to be visualized on HPSEC.

## Discussion

*G. thermodenitrificans* T12 has been shown to ferment biomass derived feedstocks with limited need for additional enzymes mainly due to its xylanolytic and pectinolytic activity and genetic accessibility [12]. The requirement for a true CBP organism to hydrolyze both hemicellulose and cellulose has not been achieved yet for *G. thermodenitrificans* and, despite major efforts to find cellulolytic *Geobacillus* spp. [8, 11–13, 23, 38], to date there is no evidence of a strain capable of efficient cellulose conversion. The endoglucanase CelA from *Geobacillus* sp. 70PC53 is currently the only characterized cellulolytic enzyme native to *Geobacillus* spp. [11, 13]. Two more *Geobacillus* derived enzymes have been proposed to be endoglucanases, M1 and M2 from *Geobacillus* sp. WSUCF1. The study describing the characterization of endoglucanases M1 and M2 shows activity of the M1 and M2 proteins against cellulose [11]. However, in this study we could not detect degradation of CMC, not even under constitutive expression of the *M1* and *M2* genes. Sequence analysis against the UniprotKB database gives highest sequence identities against peptidases and additionally, we could not identify active site residues based on sequence comparison to other endoglucanases of the GH5 family. Therefore, we assume that these enzymes are most likely peptidases and not true endoglucanases.

We screened the metagenome of 73 *G. thermodenitrificans* strains for genes that match with the HMM profiles of every glycoside hydrolase family known to contain cellulases yielding a total of 82 proteins that matched with one or more profiles. To reduce the number of false positives obtained in this approach, a more reliable cut-off threshold can be made using validated proteins of each protein family as a training set against other families. Two putative endoglucanases (GE39 and GE40) of glycoside hydrolase family 5 were identified that have been shown to be located in the HUS locus of the isolation strain. The genetic variation between the HUS locus of T12 and the partial HUS locus encoded by scaffold 9 is remarkable, as previous HUS loci comparison between three *G. thermodenitrificans* strains showed no variation in genetic content [16]. The protein architecture of GE39 contains a GH5 catalytic domain, and active site residues have been identified at positions E188 and E318. The GE39 sequence shows identity to several endoglucanases (Additional file 2: Figure S1) and structure modeling of GE39 revealed a folding similar to a GH5 exo-1,3-β-glucanase from *S. cerevisiae*. Based on structure modeling we expected GE39 to belong to the GH family 5 glucanases. However, protein alignment reveals highest identity (36%) to a GH5 β-xylosidase from *Phanerochaete chrysosporium* (r*Pc*Xyl5) [39]. For r*Pc*Xyl5 it was shown that it was most active against *p*-nitrophenyl-β-d-xylopyranoside (pNPbX) with minor activities against xylan, which is in agreement with the lack of activity seen in our activity assays on chromogenic xylan and cellulose substrates. Likewise, we found GE39 to be active against *p-*nitrophenyl-β-d-xylopyranoside. In contrast to r*Pc*Xyl5, the GE39 β-xylosidase showed side activity against *p*-nitrophenyl-β-d-glucopyranoside (pNPβX).

Where enzyme GE39 did not show glucanase activity, enzyme GE40 was demonstrated to be active against 2-HE-cellulose, CM-cellulose and barley glucan. The protein architecture of GE40 is comprised of a GH5_4 catalytic domain, an immunoglobulin-like module and a carbohydrate binding module of family 46 (Fig. 6). Family 46 CBMs, with the ability to bind cellulose and glucan, are always located on the C-terminus of enzymes containing a GH5_4 catalytic domain and an immunoglobulin (Ig)-like module. The Ig-like module is believed to act as a structural hinge, thereby holding the catalytic domain and the CBM in position for optimal enzymatic activity [36]. The CBM acts in synergy with the GH5_4 catalytic domain to bind glucans and thereby aid in the hydrolytic cleavage of the substrate [35]. Although cellulase activity was clearly demonstrated in the CFE of a *GE40* expressing *E. coli* culture, we could only detect minor activity when *GE40* was expressed in *G. thermodenitrificans* T12 (Additional file 4: Figure S2). Furthermore, SDS PAGE analysis of the cell lysate from a *GE40* expressing T12 culture did not show heterologous protein formation and no additional protein bands were visualized (data not shown). We have provided evidence that GE40 is a novel endoglucanase, however, further development of suitable promoters and/or signal peptide sequences is needed to increase protein yield and thereby the extracellular activity required for efficient cellulose conversion.

In an attempt to complement the cellulolytic machinery of *G. thermodenitrificans*, we introduced several cellulases from *C. thermocellum* into strain T12. By removing the dockerin domain we created smaller constructs that were expected to be easier to introduce to *G. thermodenitrificans* T12. The removal of the dockerin domain of CelK and CelS was shown not to reduce the enzymatic activity [40] and activity of both endoglucanases and exoglucanases from *C. thermocellum* against amorphous cellulose has been demonstrated [41]. Therefore, we hypothesize that their removal has not been instrumental in the lack of activity seen in our study. The lack of activity observed for CelS, CelSH and CelC, is possibly caused by insufficient protein production as we did not detect heterologous protein by SDS-PAGE analysis on the intracellular protein fraction (data not shown) and no intracellular or extracellular enzyme activity was detected by using chromogenic substrates and HPSEC (data not shown). We therefore hypothesize that problems at the transcription or early translational stage hamper production of functional enzymes. The fused signal peptides may also impact the efficiency, but as no clear intracellular accumulation was observed, we consider this not a major restriction at this stage. Cultures producing T12-CelK, T12-CelA and T12-GE40 did show both extracellular (Fig. 8, Additional file 4: Figure S2) and intracellular cellullolytic activity (data not shown). When *G. thermodenitrificans* strains that were demonstrated to be able to degrade cellulose were grown in liquid cultures containing CMC as sole carbon source, we were unable to detect organic acids. This observation once more indicates a lack of sufficient activity due to low expression of the enzymes and a need for further optimization.

## Conclusions

This study shows the potential of metagenome mining for the discovery of novel cellulases. Metagenome-derived enzyme GE39 was shown to be a novel GH5 β-xylosidase with some β-glucosidase activity. Enzyme GE40 was shown to be a novel GH5 endoglucanase that is active against amorphous cellulose and barley glucan and had 55% identity to its closest ortholog *Bh*Cel5b from *Bacillus halodurans*. Enzyme GE40 is the second endoglucanase retrieved from *Geobacillus* and the first found in *G. thermodenitrificans*.

We also demonstrated the ability of *Geobacillus thermodenitrificans* T12 to act as a host for heterologous cellulase expression. Although the degradation of cellulose by strain T12 still requires optimization, as activities remained low, the methods described in this study provide a starting point for further development of *Geobacillus* spp. as potential hosts for consolidated bioprocessing.

## Additional files

Additional file 1: **Table S1.** Codon harmonization of *celS* from *C****.*** *thermocellum*. An overview of the codon harmonization method used to adapt the *C. thermocellum* derived exoglucanase encoding gene *celS* for expression in *G. thermodenitrificans. (XLSX 78 kb)*

Additional file 2: **Figure S1.** Phylogenetic tree of GH5 family hydrolases. The evolutionary history was inferred by using the Maximum Likelihood method based on the JTT matrix-based model [42]. The percentage of trees in which the associated taxa clustered together is shown next to the branches. Initial tree(s) for the heuristic search were obtained automatically by applying Neighbor-Join and BioNJ algorithms to a matrix of pairwise distances estimated using the Jones Thornton Taylor (JTT) model, and then selecting the topology with superior log likelihood value. The tree is drawn to scale, with branch lengths measured in the number of substitutions per site. The analysis involved 27 amino acid sequences. All positions containing gaps and missing data were eliminated. There were a total of 198 positions in the final dataset. Evolutionary analyses were conducted in MEGA7 [43]. Open circle: eukaryotic origin; closed triangle: thermophilic organism; closed diamond: sequences obtained in this study. (PDF 12 kb)

Additional file 3: **Table S2.** Overview of measured absorbance of different chromogenic substrates after incubation with the metagenome derived putative cellulases GE39 and GE40 expressed from *E. coli*. Overview of measured absorbance of different chromogenic substrates after incubation with the metagenome derived putative cellulases GE39 and GE40 expressed from *E. coli*. The final reaction mixture in each well of the substrate plate consisted of 145 μL sodium phosphate buffer (pH 6.0) and 5 μL of CFE. Plates were then sealed using an aluminum adhesive foil and incubated at 60 °C in a rotary shaker at 180 RPM. After 24 h the reaction mixture was collected in a product plate by centrifugation (2700×g, 10 min) and absorbance was measured at 595 nm (blue) and 517 nm (red) using a plate reader (Biotek Instruments Inc., Winooski, VT, USA). Negative control consisted of sodium phosphate buffer and CFE from an *E. coli* culture containing empty pCDF1b plasmid. The thermostable endoglucanase, CelTM, (Megazyme, Wicklow, Ireland) from *Thermotoga maritima* was used as positive control (+C) at a concentration of 1 μg/mL. Values for the negative control have been subtracted. CFE of the GE40 expressing *E. coli* culture showed high activity towards cellulose and barley derived β-glucan. In contrast, CFE of the GE39 producing *E. coli* culture showed no activity to any of the chromogenic substrates. (PDF 147 kb)

Additional file 4: **Figure S2.** Congo red assays of cellulase expressing *G. thermodenitrificans* cultures. Congo red assays of *G. thermodenitrificans* cultures grown on LB2 medium with 1% carboxymethylcellulose. Each culture produces a different cellulase. Ø: empty plasmid (pNW33n) control; CelA: GH5 endoglucanase CelA (*Geobacillus* 70PC53); CelK: GH9 exoglucanase (*C. thermocellum*); GE40wt: GH5 endoglucanase (*Geobacillus* metagenome derived) containing its native signal peptide; GE40: GH5 endoglucanase (*Geobacillus* metagenome derived). (PDF 48 kb)
