# Supplementary figures and images for: Engineering Geobacillus thermodenitrificans to introduce cellulolytic activity; expression of native and heterologous cellulase genes
Source: BMC Biotechnol. 2018 Jun 27;18:42. doi: 10.1186/s12896-018-0453-y (PMC6020330; doi:10.1186/s12896-018-0453-y)

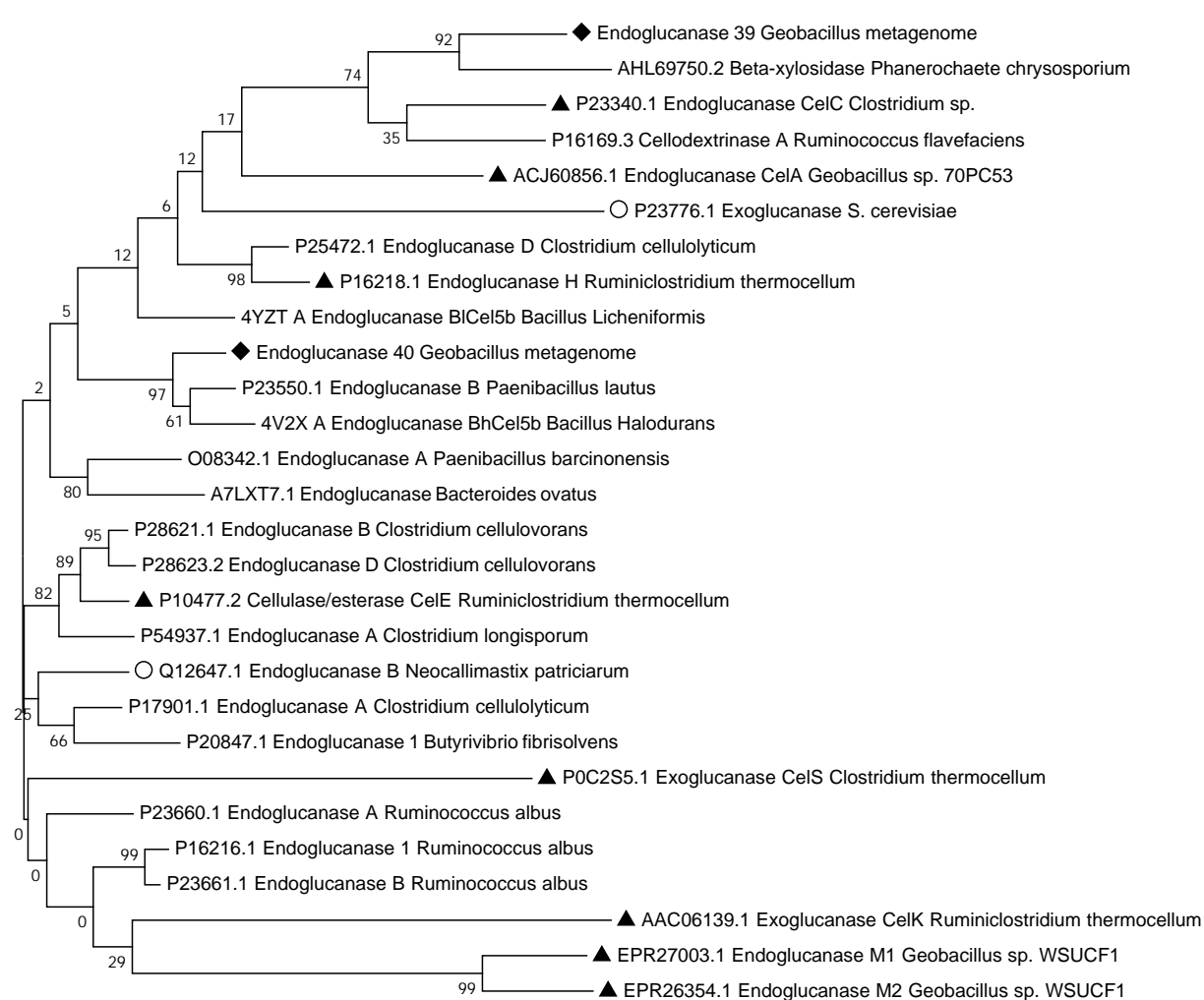

0.5

Supplement: Supplementary file 2 — Figure S1. Phylogenetic tree of GH5 family hydrolases. The evolutionary history was inferred by using the Maximum Likelihood method based on the JTT matrix-based model [42]. The percentage of trees in which the associated taxa clustered together is shown next to the branches. Initial tree(s) for the heuristic search were obtained automatically by applying Neighbor-Join and BioNJ algorithms to a matrix of pairwise distances estimated using the Jones Thornton Taylor (JTT) model, and then selecting the topology with superior log likelihood value. The tree is drawn to scale, with branch lengths measured in the number of substitutions per site. The analysis involved 27 amino acid sequences. All positions containing gaps and missing data were eliminated. There were a total of 198 positions in the final dataset. Evolutionary analyses were conducted in MEGA7 [43]. Open circle: eukaryotic origin; closed triangle: thermophilic organism; closed diamond: sequences obtained in this study. (PDF 12 kb) [file 12896_2018_453_MOESM2_ESM.pdf]

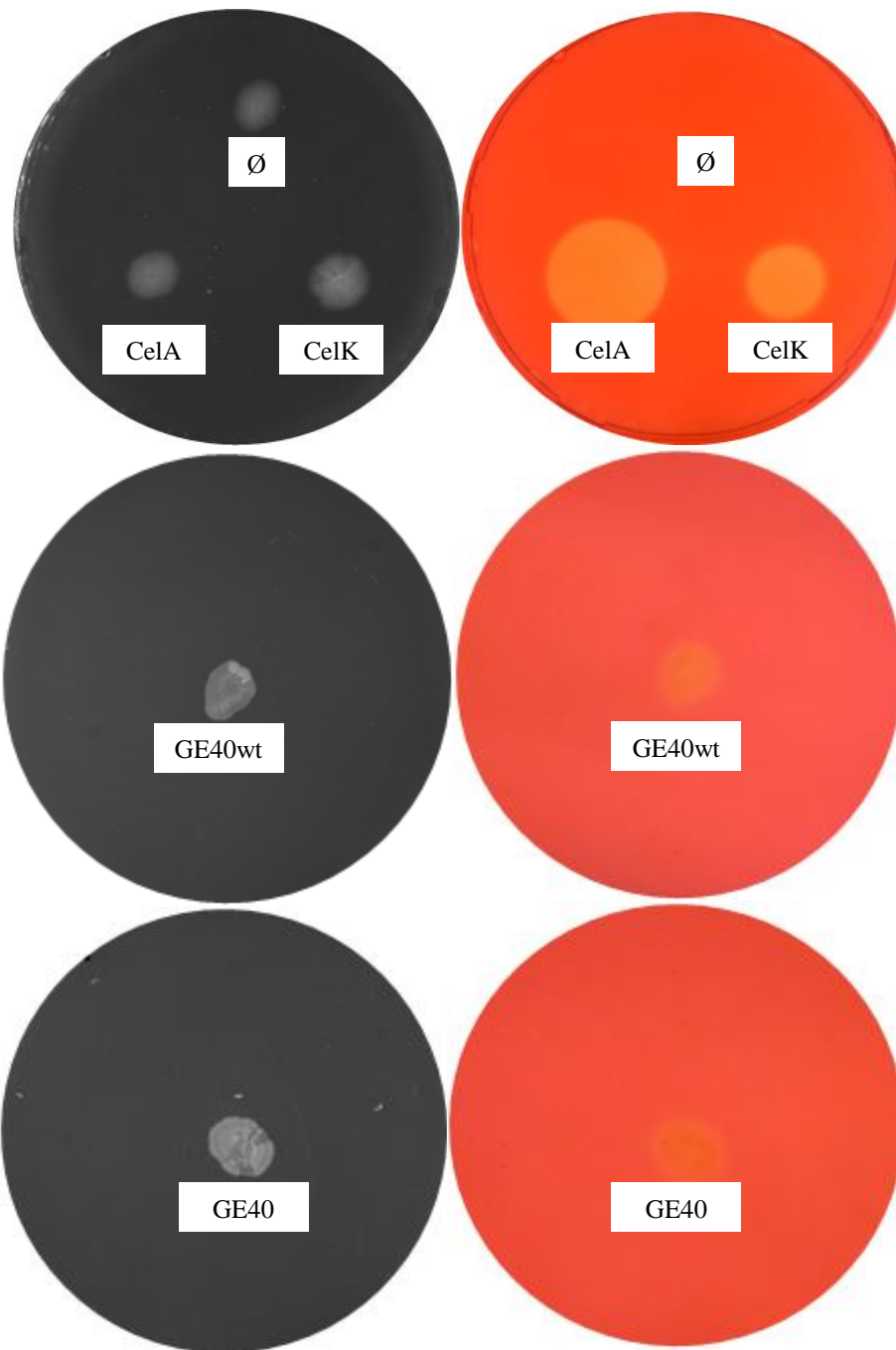

Supplement: Supplementary file 4 — Figure S2. Congo red assays of cellulase expressing G. thermodenitrificans cultures. Congo red assays of G. thermodenitrificans cultures grown on LB2 medium with 1% carboxymethylcellulose. Each culture produces a different cellulase. Ø: empty plasmid (pNW33n) control; CelA: GH5 endoglucanase CelA (Geobacillus 70PC53); CelK: GH9 exoglucanase (C. thermocellum); GE40wt: GH5 endoglucanase (Geobacillus metagenome derived) containing its native signal peptide; GE40: GH5 endoglucanase (Geobacillus metagenome derived). (PDF 48 kb) [file 12896_2018_453_MOESM4_ESM.pdf]
