# Supplementary material for: Engineering Geobacillus thermodenitrificans to introduce cellulolytic activity; expression of native and heterologous cellulase genes
Source: BMC Biotechnol. 2018 Jun 27;18:42. doi: 10.1186/s12896-018-0453-y (PMC6020330; doi:10.1186/s12896-018-0453-y)
Supplement: Supplementary file 3 — Table S2. Overview of measured absorbance of different chromogenic substrates after incubation with the metagenome derived putative cellulases GE39 and GE40 expressed from E. coli. Overview of measured absorbance of different chromogenic substrates after incubation with the metagenome derived putative cellulases GE39 and GE40 expressed from E. coli. The final reaction mixture in each well of the substrate plate consisted of 145 μL sodium phosphate buffer (pH 6.0) and 5 μL of CFE. Plates were then sealed using an aluminum adhesive foil and incubated at 60 °C in a rotary shaker at 180 RPM. After 24 h the reaction mixture was collected in a product plate by centrifugation (2700×g, 10 min) and absorbance was measured at 595 nm (blue) and 517 nm (red) using a plate reader (Biotek Instruments Inc., Winooski, VT, USA). Negative control consisted of sodium phosphate buffer and CFE from an E. coli culture containing empty pCDF1b plasmid. The thermostable endoglucanase, CelTM, (Megazyme, Wicklow, Ireland) from Thermotoga maritima was used as positive control (+C) at a concentration of 1 μg/mL. Values for the negative control have been subtracted. CFE of the GE40 expressing E. coli culture showed high activity towards cellulose and barley derived β-glucan. In contrast, CFE of the GE39 producing E. coli culture showed no activity to any of the chromogenic substrates. (PDF 147 kb) [file 12896_2018_453_MOESM3_ESM.pdf]

**Substrate**	**GE39**	**GE40**	**+C**
CPH-2-hydroxyethylcellulose	0.002	1.164	1.0705
CPH-β-glucan from barley	0.001	0.382	0.1495
CPH-β-glucan from yeast	0.019	0	0.034
CPH-curdlan	0.017	0.016	0
CPH-pachyman	0.007	0.021	0
CPH-pullulan	0.002	0.003	0
CPH-dextran	0	0.001	0.001
CPH-chitosan	0.1	0	0
CPH-xyloglucan	0.015	0.003	0.043
CPH-amylopectin	0	0.001	0
CPH-amylose	0	0.004	0.008
CPH-xylan	0	0	0.0035
CPH-arabinan	0	0	0.007
CPH-pectic galactan	0.011	0.002	0.002
CPH-galactomannan	0	0	0.1425
CPH-rhamnogalacturonan (soybean)	0	0	0.0225
